# Construction of Ultrathin Nitrogen-Doped Porous Carbon Nanospheres Coated With Polyaniline Nanorods for Asymmetric Supercapacitors

**DOI:** 10.3389/fchem.2019.00455

**Published:** 2019-06-26

**Authors:** Pingping Yu, Qunliang Wang, Lingxia Zheng, Yanfeng Jiang

**Affiliations:** ^1^Department of Electronic Engineering, College of Internet-of-Things, Jiangnan University, Wuxi, China; ^2^Department of Applied Chemistry, Zhejiang University of Technology, Hangzhou, China

**Keywords:** sustainable sources, ultrathin porous nanospheres, polyaniline nanorods, nitrogen-doped, supercapacitors

## Abstract

Porous carbon materials produced by biomass have been widely studied for high performance supercapacitor due to their abundance, low price, and renewable. In this paper, the series of nitrogen-doped hierarchical porous carbon nanospheres (HPCN)/polyaniline (HPCN/PANI) nanocomposites is reported, which is prepared via *in-situ* polymerization. A novel approach with one-step pyrolysis of wheat flour mixed with urea and ZnCl_2_ is proposed to prepare the HPCN with surface area of 930 m^2^/g. Ultrathin HPCN pyrolysised at 900°C (~3 nm in thickness) electrode displays a gravimetric capacitance of 168 F/g and remarkable cyclability with losing 5% of the maximum capacitance after 5,000 cycles. The interconnected porous texture permits depositing of well-ordered polyaniline nanorods and allows a fast absorption/desorption of electrolyte. HPCN/PANI with short diffusion pathway possesses high gravimetric capacitance of 783 F/g. It can qualify HPCN/PANI to be used as cathode in assembling asymmetric supercapacitor with HPCN as anode, and which displays an exceptional specific capacitance of 81.2 F/g. Moreover, HPCN/PANI//HPCN device presents excellent cyclability with 88.4% retention of initial capacity over 10,000 cycles. This work will provide a simple and economical protocol to prepare the sustainable biomass materials based electrodes for energy storage applications.

## Introduction

Carbon materials as the developing anodes have played pivotal roles in the energy storage area owing to its abundant sources, cost-effective, high chemical stability, and good conductivity (Gao and Fang, 2015; Benzigar et al., 2018). The excellent properties endow carbon materials (carbon nanotubes (CNT), activated carbon (AC) and graphene) with promising potential applications in the electric double-layer capacitance (EDLC) supercapacitors (Wu et al., 2017; Jiang et al., 2018). The electrochemical performances of the supercapacitors largely depend on characteristics of these adopted carbon materials, including morphologies, surface properties, specific surface area, and porous channels (Peng et al., 2014; Liu et al., 2016; Goldfarb et al., 2017; Li et al., 2018; Yang et al., 2018). Therefore, carbon materials with accessible surface area as well as high electron transport efficiency are essential for preparing the carbon-based supercapacitors. The materials with hierarchical porous nanostructure are intensively investigated recently (Badwal et al., 2014; Xu et al., 2018), which could be the promising candidate to satisfy the multiple requirements for the supercapacitors.

Activated carbon is generally prepared by pyrolyzing versatile carbonaceous precursors with physical or chemical activation to introduce the hierarchical pores. However, when the surface area increases to 3,000 m^2^ g^−1^, its electric capacity is still low (100–140 F/g) in the organic electrolyte (Barbieri et al., 2005). The interplayed aspects indicate that the capacity depends on the pore size distribution, accessibility of the electrolyte and the electrical conductivity (Zhang H. et al., 2015). Therefore, numerous biomass-based carbon combining hierarchical pores and heteroatom doping have gained considerable attentions, resulting in pesudocapacitance and electrical double layer capacitance. Carbonization of natural biomass materials, including seaweeds (Kang et al., 2015), peanut shell (Ding et al., 2015), rice bran (Hou et al., 2014), plant leaves (Liu B. et al., 2017; Zhang et al., 2018; Zhao et al., 2018), fruit (Wu et al., 2014), and wheat flour (Wu et al., 2015; Yu et al., 2016), has been investigated. It is demonstrated as a feasible approach, which could result in a low cost and eco-friendly way to prepare hierarchical porous carbon electrodes.

Among all the kinds of the investigated biomass, wheat flour is considered as the most promising one. Wheat flour can be well dispersed in aqueous solution via magnetic stirring to form suspension because it contains starch (72–80%) and protein (8–10%). Thus, wheat flour is a green carbon source to prepare the hierarchical porous carbon. Chemical activation is a preferable approach to prepare the high performance porous carbon using the chemical agents (KOH, NaOH, ZnCl_2_ and H_3_PO_4_) (Duan et al., 2016; Lei et al., 2016; Pang et al., 2016a,b; Goldfarb et al., 2017; Yang et al., 2019). The strong activation effect of KOH and NaOH can degrade the wheat flour into small molecular and lose a large proportion of carbon atoms, making the low yield of carbon materials (Wang and Liu, 2017). ZnCl_2_ as a milder activation reagent can react with precursor by dehydrating and cross-linking along the temperature increase, creating hierarchical pores, high specific surface area and high yield.

Polyaniline (PANI) is considered as a good developing supercapacitor electrode due to its large pseudocapacitance, intrinsic redox states and simple synthesis (Yu et al., 2013a, 2014, 2017). However, because of the degradation of PANI electrodes in the process of repeated charging and discharging, the cycle life of PANI electrodes is usually poor (Liu et al., 2014). In order to overcome this shortcoming, combination of the well-ordered PANI nanostructure and carbon materials has been explored to prevent the collapse. Wang et al. reported that the ordered whiskerlike PANI/mesoporous carbon composites formed “V-type” channels which shorten the ion transport pathway and increase the contacted area for electrolyte (Wang et al., 2006). Liu et al. synthesized hierarchical graphene/PANI hollow microspheres hybrid electrode with supercapacitor capacitance of 446.2 F/g, which exhibits an outstanding long cycle life (Liu L. et al., 2017). Sulfur-encapsulated porous carbon nanospheres/polyaniline composites were synthesized to improve chemical stability and electronic conductivity (Li et al., 2016). However, these PANI-based binary composites exhibit single-scale pores, complex synthetic procedure, and high cost, which is not suitable for the high-performance-supercapacitor.

**Figure 1:**
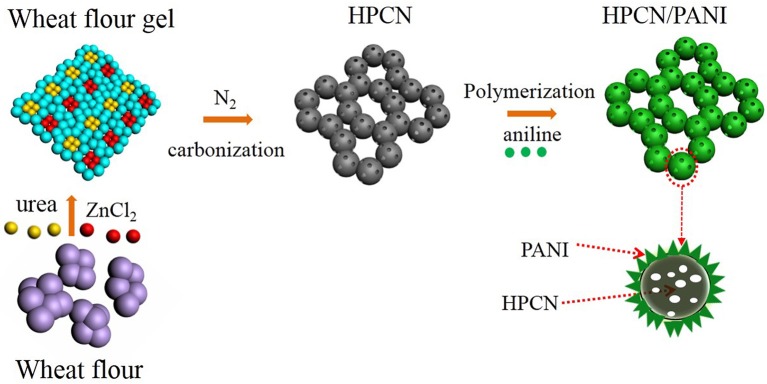
Schematic illustration of fabricating HPCN/PANI composites.

In this study, the one-step carbonization for wheat flour is employed by pyrolysis of urea and ZnCl_2_ to prepare interconnected hierarchical porous N-doped carbon nanospheres (HPCN). Figure 1 presents the approach for the fabrication of the HPCN/PANI composites by chemical oxidative polymerization. The highest specific capacitance of HPCN with surface area of 930 m^2^/g is 168 F/g, and then PANI nanorods are vertically coated on the HPCN by chemical bonding interaction with nitrogen groups, providing the significant enhancement of supercapacitor performances. Moreover, this assembled asymmetric supercapacitor (ASC) combining HPCN and HPCN/PANI can work in 1 M H_2_SO_4_ electrolyte and its electrochemical performances are investigated in this paper.

## Experimental Sections

### Synthesis of Hierarchical Porous Hpcn

HPCN were formed using wheat flour as renewable biomass resource through one-step carbonization. Typically, the waste wheat flour, urea and ZnCl_2_ (1:1:2, w/w) were added to 50 mL distilled water with magnetic stirring, followed by carbonization at 700–900°C for 1 h under N_2_ flow. As-carbonized samples were stirred in 10% HCl and DI water successively for 15 h. After purification, the HPCN was dried under 80°C vacuum oven. The obtained products were termed as HPCN7, HPCN8, and HPCN9 standing for the pyrolysis temperature at 700, 800, and 900 °C, respectively.

### Fabrication of HPCN/PANI

The HPCN (100 mg) were dispersed in 1 M H_2_SO_4_ solution containing aniline monomers (AN, 45.65 μL) by strong stirring. 40 mL 1 M H_2_SO_4_ of ammonium persulfate (APS, 114.12 mg, [AN]/[APS] = 1:1) solution was rapidly added with stirring for more than 5 min. The oxidative reaction was conducted at −5°C overnight, and the obtained composites were filtered, rinsed with DI water and overnight dried at 50°C. The content of PANI is 26.21% in HPCN7/PANI, 29.34% in HPCN8/PANI and 32.05% in HPCN9/PANI, respectively.

### Material Characterization

The composites were conducted with a field-emission scanning electron microscope (FESEM, Zeiss Sigma), a transmission electron microscope (TEM, TECNAI G^2^ S-TWIN), X-ray diffraction (XRD, Bruker D8-A25, λ = 1.5405 Å, Cu Kα radiation, 10-50°), Raman spectra (LabRam-1B, 632.8 nm laser), X-ray photoelectron spectroscopy (XPS, PHI 5000C ESCA) and ASAP 3000.

### Electrochemical Measurements

The mixture was blended with ratio of 90/5/5 for as-prepared samples/carbon black/ polytetrafluoroethylene. The uniform paste was coated on a titanium mesh (1 cm in diameter) as working electrode. The HPCN and HPCN/PANI were tested in three-electrode cell. The asymmetric supercapacitors were fabricated with negative electrode of HPCN9 and positive electrode of HPCN9/PANI. The filter paper were placed between two electrodes to be the separator. The mass of active materials deposited on Ti mesh was approximated to be 2.0 mg. Electrochemical performances of electrodes were conducted with an electrochemical workstation of CHI 660D in electrolyte of 1 M H_2_SO_4_ aqueous.

According to the GCD plots, the specific capacitance of the HPCN and HPAN/PANI electrodes can be calculated depending on equation (1):

(1) $$\begin{array}{r} C_{sp}=\frac{I\Delta t}{m\Delta V} \end{array}$$

where I (A) is the current, Δt (s) stands for the time required for full discharge, m (g) is the weight of the electrodes, and ΔV (V) is the voltage difference. In order to keep the charge balance, the mass ratio of positive and negative electrode was calculated using the following formula (2):

(2) $$\begin{array}{r} \frac{m_{+}}{m_{-}}=\frac{C_{-}V_{-}}{C_{+}V_{+}} \end{array}$$

The specific capacitance (C_asy_), energy density (E, Wh/kg) and power density (P, W/kg) of ASC device were obtained by the following equations:

(3) $$\begin{array}{r} C_{asy}=\frac{I\Delta t}{M\Delta V} \end{array}$$

(4) $$\begin{array}{r} \text{E}=\frac{C_{asy}{\Delta V}^{2}}{2\times 3.6} \end{array}$$

(5) $$\begin{array}{r} \text{P}=3600\times \frac{E}{\Delta t} \end{array}$$

where M (M = m_+_+m_−_) is the total mass of electrode materials.

## Results and Discussion

**Figure 2:**
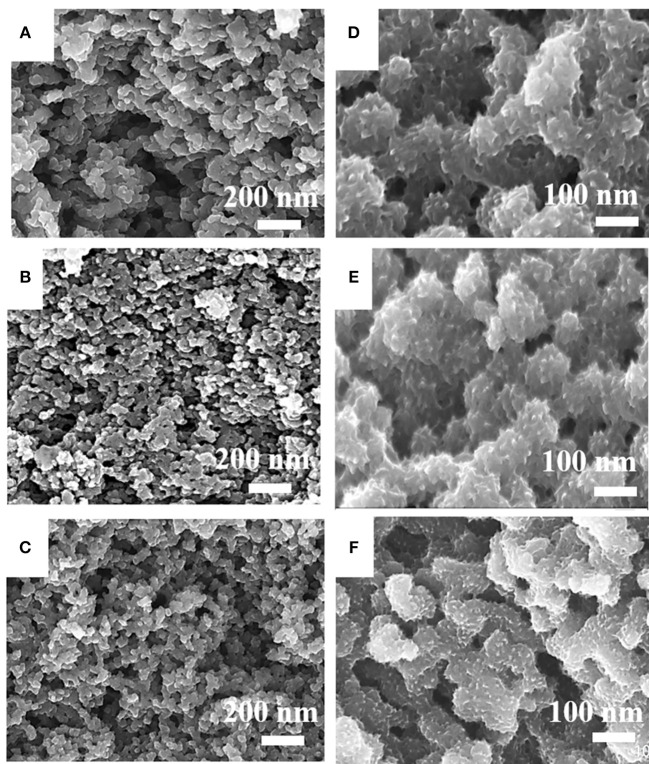
FESEM images of HPCN7 **(A)**, HPCN8 **(B)**, and HPCN9 **(C)**, HPCN7/PANI **(D)**, HPCN8/PANI **(E)** and HPCN9/PANI **(F)**.

The morphologies of HPCN7, HPCN8, and HPCN9 (carbonized at 700, 800 and 900°C) indicate that the samples consist of a large amount of uniformly interconnected carbon nanospheres (Figures 2A–C). The diameter of the HPCN7 (Figure 2A) is 70 nm, which is higher than that of HPCN8 (61 nm, Figure 2B) and that of HPCN9 (50 nm, Figure 2C) because carbonization temperature increases from 700 to 900°C. In this chemical activation process, ZnCl_2_ as dehydrating agent can promote the condensation reaction of wheat flour, reduce the gasification of carbon atoms and lead to high carbon yields (>35%). ZnCl_2_ is also as an effective activation-graphitization agent that promotes aromatic condensation reactions at high temperature due to its Lewis acid nature and introduce the porous structure to improve the surface area. The nanospheres are interconnected to form the porous network. This structure has benefit for fast electrode/electrolyte interface kinetics.

The porous contour of the HPCN can be used as a buffer boost for expansion and shrinking of PANI nanorods. PANI nanorods are vertically deposited on the HPCN7, HPCN8, and HPCN9 to improve the specific capacitance, as shown in Figures 2D–F. Compared to HPCN9/PANI (Figure 2F), there are obvious denser and longer PANI nanorods stacked on HPCN7/PANI (~50 nm, Figure 2D) and HPCN8/PANI (~40 nm, Figure 2E) than those in HPCN9/PANI. This shows that the thicknesses of the PANI layers of HPCN7/PANI and HPCN8/PANI are higher than that of the HPCN9/PANI. This may be due to the HPCN7 and HPCN8 with more nitrogen-groups and higher specific surface area to be bonded with much more aniline monomers to form the PANI nanorods.

**Figure 3:**
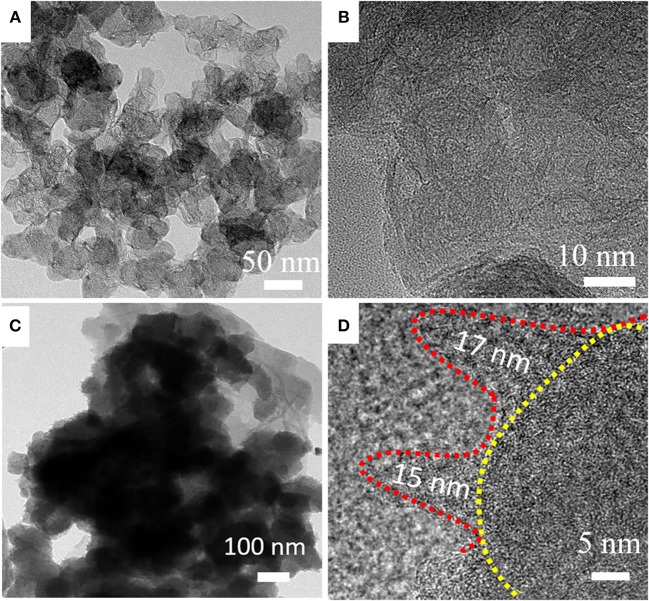
Different magnification for TEM images of HPCN9 **(A,B)** and HPCN9/PANI **(C,D)**.

The above analysis shows that HPCN9/PANI has thinner PANI layer. The average diameter of carbon nanospheres in HPCN9 is 50 nm. Their thin carbonaceous walls are connected to form the reticular morphology (Figure 3A). Plenty of nanopores can be observed in the HPCN9. The thickness of these carbonaceous walls (Figure 3B) is about 3 nm equal to 6–8th layer of graphene. For the sample HPCN9/PANI (Figure 3C), the PANI nanorods are grown on the porous carbon nanospheres, maintaining the interconnected network structure. Figure 3D presents that the diameter and the length of the PANI nanorods are about 5–10 and 15–17 nm, respectively. The well-ordered and much smaller sized PANI in HPCN9/PANI can largely short the diffusion pathway by enhancing utilization of the active materials, improving the electrochemical performances.

**Figure 4:**
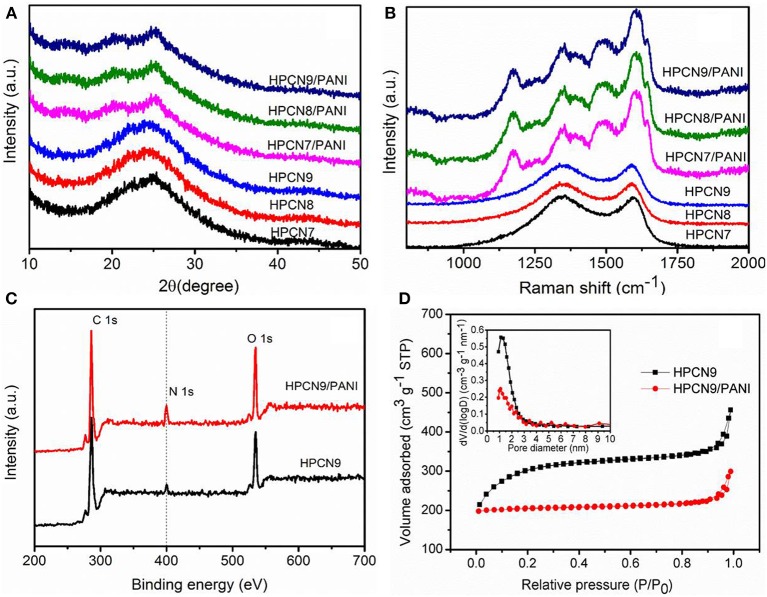
**(A)** XRD patterns of HPCN7, HPCN8, HPCN9, HPCN7/PANI, HPCN8/PANI, and HPCN9/PANI composites. **(B)** Raman spectra for all the samples. XPS spectra **(C)** and N_2_ sorption isotherm **(D)** (inset: pore size distribution) of HPCN9 and HPCN9/PANI.

Figure 4A shows XRD profiles of the HPCN and HPCN/PANI composites. As-prepared HPCN7, HPCN8, and HPCN9 all show two characteristic diffraction peaks (002) and (101) at around 24° and 44°, respectively. The (002) diffraction with broad width and low intensity of (101) plane indicate the amorphous carbon structure (Yu et al., 2016). Three crystalline peaks located at 2θ = 14.8°, 25.2°, and 20.3° for the three-HPCN/PANI composites are assigned to be the crystal planes of (011), (200), and (020) for emeraldine salt polyaniline, indicating the periodicity of the perpendicular and the parallel of the polymer chains, respectively, (Yu et al., 2013a).

The Raman spectra of HPCN7, HPCN8, and HPCN9 (Figure 4B) exhibit the strong D-peak at 1,344 cm^−1^, representing disordered carbon confirmed with XRD results (Kudin et al., 2008). G-band at 1,597 cm^−1^ indicates graphitic carbon with vibration of sp^2^ C. The value of I_D_/I_G_ for HPCN7, HPCN8, and HPCN9 is 1.04, 1.03, and 1.01, respectively. It indicates that the HPCN9 has relatively higher graphitized degree, offering good electric conductivity. There are other additional four characterization peaks for HPCN7/PANI, HPCN8/PANI, and HPCN9/PANI. The peak at 1,175 and 1,482 cm^−1^ is assigned to in-plane C-H bending and C = N stretching of quinoid ring, respectively. At 1356 and 1,593 cm^−1^, the peaks indicate protonated C-N stretching vibration and C-C stretching of benzenoid ring. It shows PANI nanorods in the emeraldine form (Yu et al., 2013a,b; Chen et al., 2016; Zheng et al., 2016).

The XPS spectra demonstrate the fact that the nitrogen atoms exist in the HPCN9 and HPCN9/PANI in Figure 4C observed the C, N, and O 1s peaks. The deconvoluted C 1s peaks of HPCN9 reveal 284.7 eV of the sp^2^ C = C bond of graphitic carbon and 285.8 eV of sp^3^ C-C/C-H bonds while the one at 287.1 eV attributes to C = O/N-C = N bonds (Figure S1a). The concentration of nitrogen species is 2.4 at.% and 6.7 at.% for HPCN9 and HPCN9/PANI, respectively (Tables S1, S2). The N 1s spectra (Figures S1b,c) of HPCN9 are deconvoluted into three bands: 401.2, 399.8 and 398.6 eV, which is corresponding to quaternary N, pyrrole N and pyridine N, respectively (Li et al., 2015; Wang et al., 2015). It indicates the O/N functional groups originated form wheat flour and urea during the carbonization process. For HPCN9/PANI, three peaks centered at 401.3, 399.6, and 398.2 eV indicate the positively charged nitrogen atoms (-NH^+^ =), benzenoid amine (-NH-) and quinoid amine (-N =) in the deconvoluted N 1s spectra (Figure S1d), respectively (Yu et al., 2013a; Zhang Y. et al., 2015; Wang and Liu, 2017).

Figure 4D is the N_2_ adsorption/desorption isotherms of the HPCN and HPCN/PANI. It can be seen that the isotherms of HPCN and HPCN/PANI are I/IV type adsorption/desorption. At the relative low pressure, there is a fast and distinct adsorption, while it shows slight hysteresis loop at P/P_0_ of 0.3–1.0. This suggests that HPCN and HPCN/PANI contains micropores and mesopores. In addition, the increment in adsorption quantity at P/P_0_ of 1.0 is caused by the small amount of macropores. The specific surface area (Figure S2) follow the trend: HPCN9 (978 m^2^/g) > HPCN8 (965 m^2^/g) > HPCN7 (930 m^2^/g). HPCN9/PANI has superior surface area of 639 m^2^/g to HPCN7/PANI (580 m^2^/g) and HPCN8/PANI (602 m^2^/g), which is higher than the reported carbon materials and PANI hybrids (Liu L. et al., 2017; Yu et al., 2017), promising to achieve favorable capacitor performance. The decreased pore volumes and specific surface area suffer from pore blockage after coating PANI nanorods by the *in-situ* polymerization, while the average pore diameter of HPCN9/PANI is still 1.0 nm (inset in Figure 3D).

**Figure 5:**
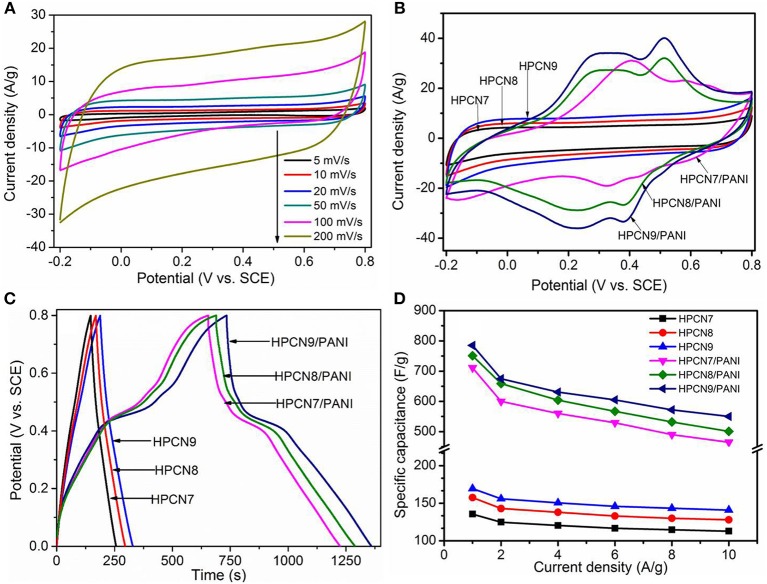
CV curves **(A)** of HPCN9 at various scan rates. Supercapacitor behavior of all samples. **(B,C)** and **(D)** shows compared CV curves, GCD response and change in specific capacitance *vs*. current density of HPCN7, HPCN8, HPCN9, HPCN7/PANI, HPCN8/PANI, and HPCN9/PANI composites.

The electrochemical characterizations of the HPCN and HPCN/PANI electrodes are demonstrated by CV curves, GCD and EIS in a three-electrode setup based on the −0.2–0.8 V. Figure 5A presents the nearly rectangular CV curves of HPCN9 electrode with scanning rate of 5–200 mV/s. There is a slight distortion because of the interconnected hierarchical porous nanospheres and nitrogen atoms doping from urea, indicating the good rate performance. It exhibits the electrochemical double layer capacitive features. The CV curve of HPCN9 possesses the higher integrated areas than those of HPCN7 and HPCN8 at 100 mV/s (Figure 5B), revealing the higher charge storage capabilities of HPCN9. Therefore, the hierarchical porous HPCN can be the good scaffold for the PANI nanorods loading. The enhanced area of CV curves is attributed to the well-ordered porous HPCN/PANI structure. Two new pairs of intense peaks in HPCN7/PANI, HPCN8/PANI, and HPCN9/PANI are assigned to the redox reactions of PANI, implying the main chain change of PANI as semiconducting leucoemeraldine/conducting polaronic emeraldine and faradaic transformation of emeraldine/pernigraniline, respectively (Yu et al., 2013b). The HPCN9/PANI shows the higher specific capacitance than HPCN7/PANI and HPCN8/PANI based on the areas of the CV curves, consistent with the GCD plots (Figure 5C). Different from the symmetric triangle plot for HPCN, there are voltage plateau and longer discharge time in the GCD plots of HPCN/PANI at 1 A/g, suggesting the pseudocapacitive characteristics. Obviously, the HPCN9/PANI possesses the optimal capacitive performance owing to the longer discharge time. Therefore, capacity performance of electrodes increases with the addition of PANI nanorods confirming by the GCD and CV tests. The maximum specific capacitance (C_sp_) of HPCN9, HPCN8, and HPCN7 obtained in Figure 5D is 168 F/g, 133 F/g, and 152 F/g, superior to the capacitance of previous published biomass-carbon electrodes (Zhang H. et al., 2015; Zhang Y. et al., 2015; Lei et al., 2016; Pang et al., 2016a,b). The HPCN9 shows the interconnected porous structure to help reducing the diffusion pathway of electrolyte ions and providing low-resistance to enhanced capability performances. The calculated C_sp_ of HPCN9/PANI, HPCN8/PANI, and HPCN7/PANI (Figure 5D) at 1 A/g is 783 F/g, 751 F/g and 710 F/g, respectively. Table 1 is the comparison of specific capacitances of HPCN/PANI and previously reported carbon/PANI composites. The exceptional capacitance mainly arises from the synergic effect of pseudocapacitance and double-layer capacitance, due to the well-ordered PANI nanorods on the interconnected porous network structure. The nanometer size of PANI with “V-type” channels facilitates the large accessible surface area between electrolyte and active species for fast faradaic reactions and shorten diffusion pathway to ensure the effective utilization of PANI nanorods.

**Table 1:** Comparison of the performances for hybrid PANI electrodes in aqueous electrolyte.

**Materials**	**C_**sp**_ (F/g)**	**Weight (mg)**	**Current density**	**System**	**Cycle life**	**References**
PANI/CNT	403.3	NA	1 A/g	3-electrode GCD	NA	Wu et al., 2017
Grapheme/PANI	446.2	NA	5 mV/s	2-electrode CV	88.7% (5,000 cycles at 2 A/g)	Liu L. et al., 2017
MnFe_2_O_4_/graphene/PANI	241	1.3	0.5mA/cm^2^	3-electrode GCD	NA	Sankar and Selvan, 2015
Porous grapheme/PANI film	740	0.82	0.5 A/g	3-electrode GCD	87% (1,000 cycles at 10A/g)	Wang et al., 2015
Kenaf-carbon/PANI	628	2	0.1 A/g	3-electrode GCD	89.2% (1,000 cycles at 0.1A/g)	Lu et al., 2016
N-Graphene/PANI	514.3	7.4	1 A/g	3-electrode GCD	87.1% (1,000 cycles at 10 A/g)	Zou et al., 2018
Macroporous carbon/PANI	662	~2.5	1 A/g	3-electrode GCD	83% (7,000 cycles at 2 A/g)	Li et al., 2015
MoS_2_/rGO/PANI	570	4-5	1 A/g	3-electrode GCD	78.6% (500 cycles at 1 A/g)	Bai et al., 2018
HPCN9/PANI	783	~2.0	1 A/g	3-electrode GCD	89.3% (5,000 cycles at 1 A/g)	This work

**Figure 6:**
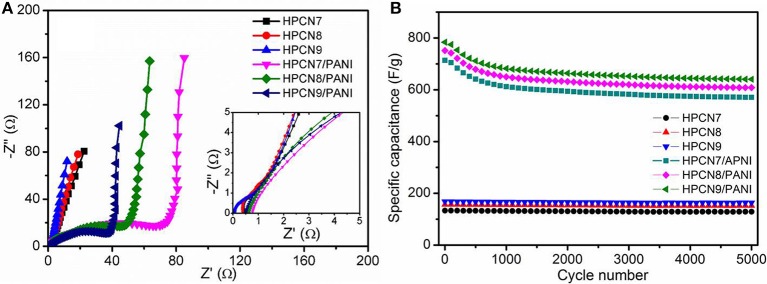
Impedance spectra of HPCN and HPCN/PANI electrodes measured from 10^5^ Hz^−^10^−2^ Hz **(A)** (inset: high frequency region). Capacitance retention of HPCN and HPCN/PANI electrodes over 5,000 cycles at 1 A/g **(B)**.

Figure 6A shows the Nyquist plots for HPCN and HPCN/PANI electrodes. HPCN7/PANI, HPCN8/PANI, and HPCN9/PANI electrodes exhibit small charge transfer resistance (R_ct_) corresponding to equivalent circuit (Figure S3), which is 0.75, 0.68, and 0.51 Ω, respectively, higher than that of HPCN7 (0.42 Ω), HPCN8 (0.32 Ω), and HPCN9 (0.17 Ω), due to the PANI nanorods grown on the porous carbon nanospheres. The decreased R_ct_ from HPCN7/PANI to HPCN9/PANI is attributed to the increased electrode/electrolyte interface area, facilitating the fast redox reactions in the as-prepared electrodes. Moreover, the almost straight line at low frequency of HPCN9/PANI plot shows ideal capacitive characteristic, indicating to the good rate performance. Apart from the high specific capacitance and small resistances, the HPCN, and HPCN/PANI electrodes also exhibit good cycling durability (Figure 6B). HPCN electrodes exhibits outstanding cycling behavior with losing 5% of initial capacitance over 5,000 cycles. HPCN9/PANI electrode remains 698 F/g and holds 89.3% of initial value after 5,000 cycles, while HPCN7/PANI and HPCN8/PANI show the capacitance retention of 85.1 and 86.5%, respectively. The fast capacitance decay may be caused by the collapse and swelling of PANI structure because of the doping/dedoping of electrolyte ions.

**Figure 7:**
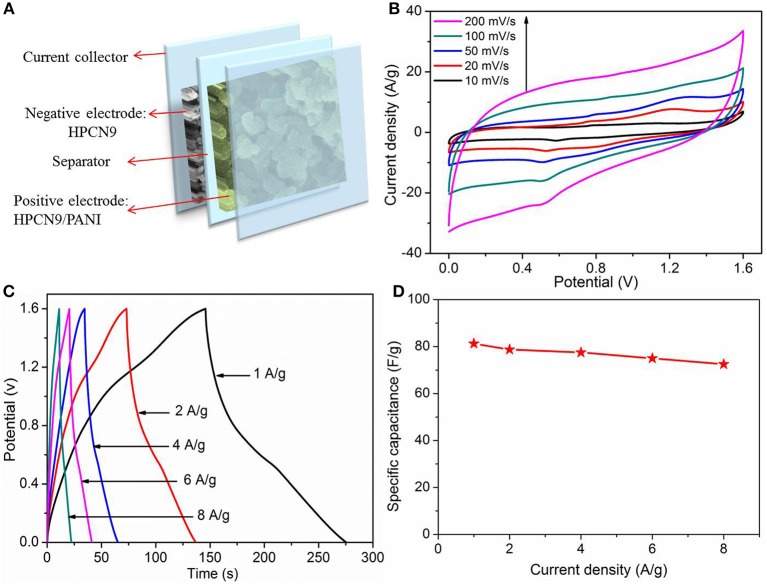
Schematic illustration of HPCN9//HPCN9/PANI asymmetric device **(A)**. CV profiles of HPCN9//HPCN9/PANI ASC device at various scan rates **(B)**. GCD profiles of HPCN9//HPCN9/PANI ASC device **(C)**. Specific capacitance of HPCN9//HPCN9/PANI ASC device vs. current densities **(D)**.

Furthermore, the HPCN9//HPCN9/PANI asymmetric supercapacitor (ASC) was fabricated by sandwiching a H_2_SO_4_/filter paper as the separator between negative (HPCN9) and positive electrode (HPCN9/PANI) (Figure 7A). The voltage window of HPCN9//HPCN9/PANI ASC device is 1.6 V. CV curves of HPCN9//HPCN9/PANI ASC device (Figure 7B) maintain the good roughly rectangular shape at 200 mV/s. The GCD curves (Figure 7C) exhibit a slight non-linearity and almost symmetric characteristic especially at the low current density for ASC device, indicating the contribution of the redox reaction from PANI. The equivalent series resistance (ESR) of ASC device is 1.4 Ω (Figure S4), indicating a small contact resistance. The C_sp_ of HPCN9//HPCN9/PANI asymmetric supercapacitor is 81.2 F/g at 1 A/g (Figure 7D), and remains 69.5% retention at 8 A/g.

**Figure 8:**
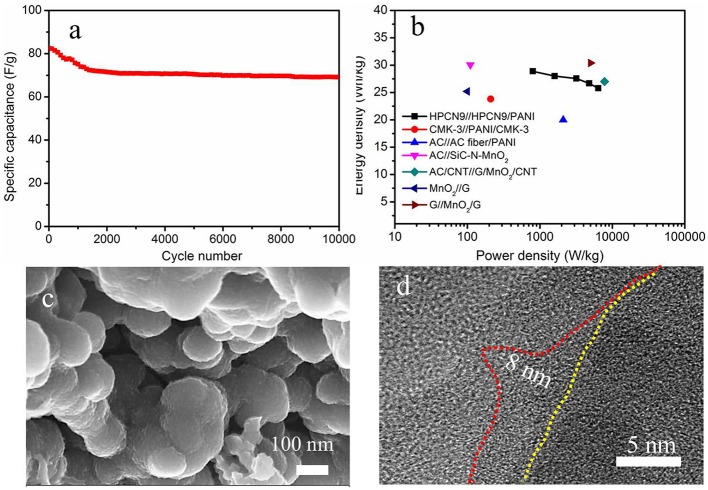
**(a)** Cycling performance after 10,000 cycles and **(b)** Ragone plots for HPCN9//HPCN9/PANI ASC device and other devices in the literatures. **(c)** SEM image and **(d)** high-resolution TEM image of HPCN9/PANI in ASC device after 10,000 cycling.

To examine the long-term cycling stability of HPCN9//HPCN9/PANI ASC, GCD tests are measured to the change of specific capacitance at 1 A/g (Figure 8a). HPCN9//HPCN9/PANI ASC shows 88.4% of initial capacitance after 10,000 cycling, lower than that of honeycomb-like porous carbon (HPC)//HPC/PANI (91.6% retention after 5,000 cycles) (Yu et al., 2016), due to the mechanical degradation of PANI in the acid electrolyte. However, the value is higher than graphene/PANI//graphene/RuO_2_ ASC (30% decay over 2500 cycles) (Zhang et al., 2011) and activated graphene//PANI/MnO_2_/carbon cloth ASC (30% loss of initial value after 5,000 cycles) (Zhao et al., 2015). Figure 8b displays the Ragone plot of HPCN9//HPCN9/PANI ASC, which has the highest energy density of 31.2 Wh/kg at 860 W/kg and retains that value of 25.1 Wh/kg at 6.88 kW/kg. As far as authors know, the results are higher than recently reported AC//AC fiber/PANI (20 Wh/kg, 2.1 kW/kg) (Salinas-Torres et al., 2013), AC//SiC-N-MnO_2_ (30.06 Wh/kg, 0.11 kW/kg) (Kim and Kim, 2014), mesoporous carbon/PANI (23.8 Wh/kg, 0.21 kW/kg) (Cai et al., 2010), AC/CNT//graphene/MnO_2_/CNT (27 Wh/kg, 7.8 kW/kg) (Cheng et al., 2013), graphene//MnO_2_ (25.2 Wh/kg, 0.1 kW/kg) (Cao et al., 2013), and MnO_2_/graphene// grapheme (30.4 Wh/kg, 5 kW/kg) (Wu et al., 2010). Furthermore, the morphology of ASC device is investigated after 10,000 cycles. The nanospheres of HPCN9 (Figure S5) still stack with each other and form a porous network structure after 10,000 cycles. Obviously, HPCN9/PANI (Figure 8c) remains well-constructed porous network structure with advantage for high efficient ion diffusion. The length of PANI nanorods decreases to be ~8 nm owing to the degration during the long cycling process (Figure 8d). The excellent electrochemical performances of HPCN9//HPCN9/PANI ASC arise from the unique structure of well-ordered PANI nanorods grown on the hierarchical interconnected porous HPCN. Nitrogen heteroatom in HPCN enhance the surface wettability and active sites for electrolyte ions. The micropores in HPCN9 can offer high available interfacial areas; the mesopores make ions with high mobility to drift into the internal regions by efficient diffusion channels, while the interconnected network provides the macropores as the ion-buffering reservoirs, contributing to the excellent rate capacity. Moreover, the nanoscale PANI with high pseudocapacitance enhances supercapacitor performances of the integrated asymmetric device. Therefore, the synergistic effect of PANI and HPCN results in high electrochemical performances.

## Conclusions

In summary, the interconnected hierarchical porous N-doped HPCN with high conductivity is successfully synthesized in this paper, starting from the easy accessible raw materials, such as sustainable wheat flour, urea and ZnCl_2_. The prepared HPCN exhibits high specific surface area of 930 m^2^/g with rational micro-mesopore distribution, which also delivers a specific capacitance of 168 F/g with remarkable stability (5% decay of initial value over 5,000 cycles). The well-ordered PANI nanorods deposited on HPCN9 displays high specific capacitance of 783 F/g. As-fabricated HPCN9//HPCN9/PANI ASC device delivers specific capacitance of 81.2 F/g, maximum energy density and power density, and 11.6% loss of original capacitance over 10,000 cycles. This work will provide a possibility of sustainable biomass materials to be the supercapacitor electrodes using the facile and low-cost process in the modern society.

## Data Availability

All datasets generated for this study are included in the manuscript/Supplementary Files.

## Author Contributions

PY designed the work and wrote the paper. QW performance the experiments. LZ polished the english of this manuscript. YJ was responsible for the drafting.

### Conflict of Interest Statement

The authors declare that the research was conducted in the absence of any commercial or financial relationships that could be construed as a potential conflict of interest.
