# Supplementary material for: Construction of Ultrathin Nitrogen-Doped Porous Carbon Nanospheres Coated With Polyaniline Nanorods for Asymmetric Supercapacitors
Source: Front Chem. 2019 Jun 26;7:455. doi: 10.3389/fchem.2019.00455 (PMC6606993; doi:10.3389/fchem.2019.00455)
Supplement: Supplementary file 1 [file Data_Sheet_1.PDF]

Supplementary Material

Table S1. XPS data of HPCN9 and HPCN9/PANI.

	C (atom%)	O (atom%)	N (atom%)
HPCN9	85.78	11.81	2.41
HPCN9/PANI	82.33	10.94	6.73

Table S2. Element analyses data of HPCN9 and HPCN9/PANI.

Elements	C (%)	H (%)	N (%)
HPCN9	82.36%	2.698%	2.361%
HPCN9/PANI	80.24%	3.123%	6.824%

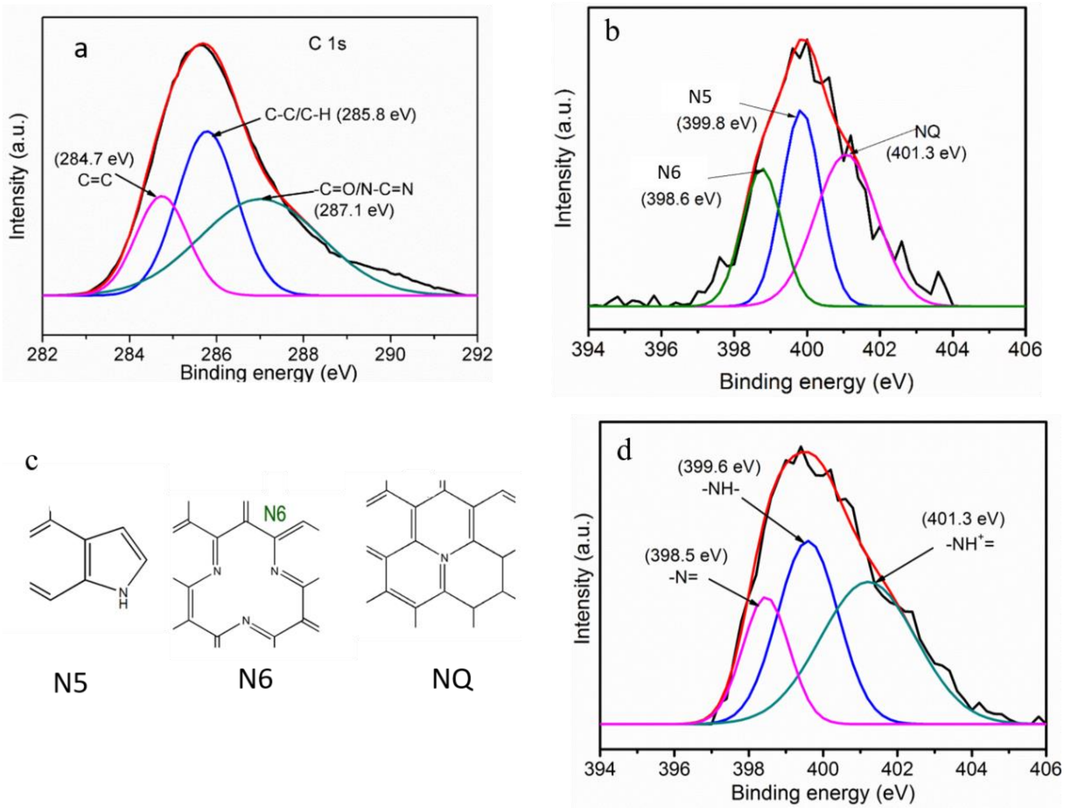

Figure S1. High resolution C 1s (a) and N 1s (b) spectra of HPCN9, (c) three types of nitrogen species in nitrogen-doped HPCN9, (d) high resolution N 1s spectra of HPCN9/PANI.

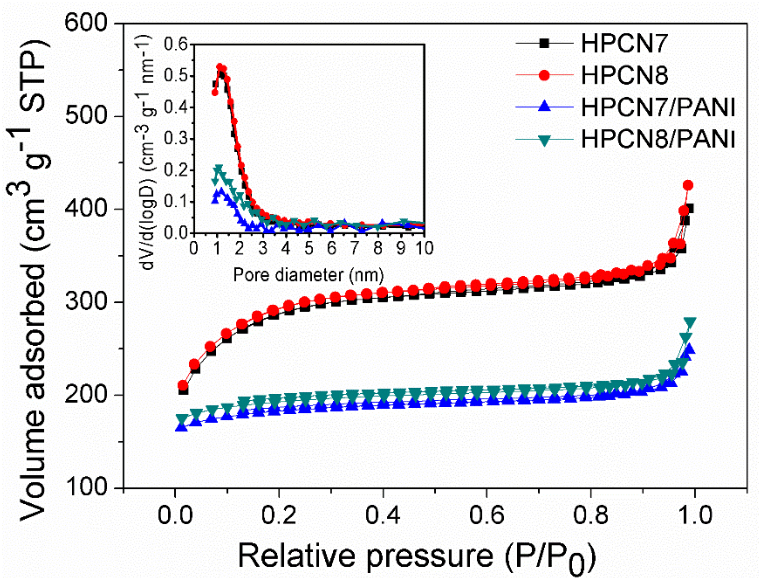

Figure S2. Nitrogen adsorption/desorption isotherm (inset is pore size distribution) of HPCN7, HPCN8, HPCN7/PANI and HPCN8/PANI.

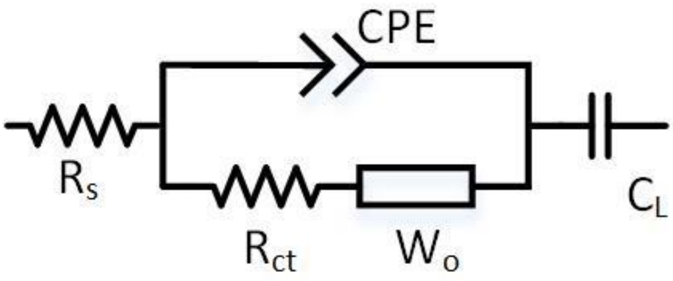

Figure S3. The equivalent circuit of the fitted Nyquist plots.

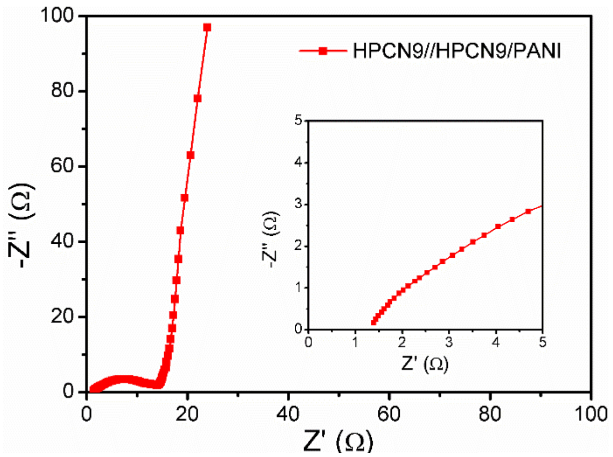

Figure S4. Nyquist plot of HPCN9//HPCN9/PANI ASC device in the frequency range of 100 kHz-0.01 Hz. The inset is the magnified view.

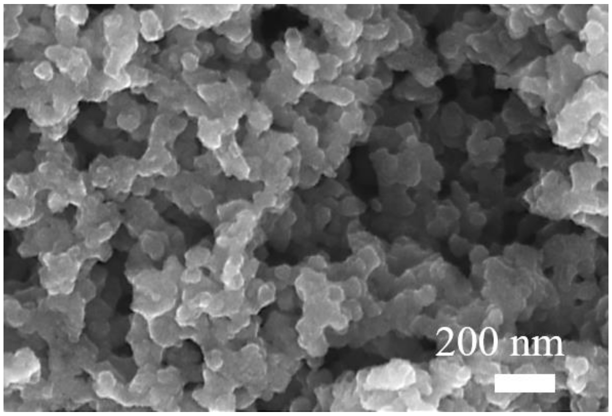

Figure S5. SEM image of HPCN9 in ASC device after 10000 cycles test.
